# Ethyl 3,3,3-trifluoro-2-hy­droxy-2-(5-meth­oxy-1*H*-indol-3-yl)propionate

**DOI:** 10.1107/S1600536811021489

**Published:** 2011-06-11

**Authors:** Zukhra Kadirova, Samat Tolipov, Oleg Fedorovskiy, Ibragimov Bakhtiyar, Nusrat Parpiev

**Affiliations:** a Tashkent Chemical–Technological Institute, Navoi St. 32, Tashkent 100011, Uzbekistan; b Institute of Bioorganic Chemistry, Mirzo-Ulugbek St. 83, Tashkent 100125, Uzbekistan; c A.N. Nesmeyanov Institute of Organoelement Compounds, Russian Academy of Sciences, Vavilova St. 28, Moscow 119991, Russian Federation; d National University of Uzbekistan named by Mirzo Ulugbek, Chemical Faculty, Vuzgorodok, Tashkent 100123, Uzbekistan

## Abstract

In the title compound, C_14_H_14_F_3_NO_4_, the 3,3,3-trifluoro­pyruvate fragment has a *syn* configuration and is noncoplanar with the indole plane [dihedral angle = 84.87 (5)°]. In the crystal, mol­ecules form inversion-related dimers *via* pairs of inter­molecular O—H⋯O hydrogen bonds. These dimers are connected by inter­molecular N—H⋯O=C(CF_3_) hydrogen bonds to form a two-dimensional network structure.

## Related literature

For background on the synthesis and activity of trifluoro­pyruvates of indole, see: Nakamura *et al.* (2008 ▶); Abid *et al.* (2008 ▶). For the crystal structures of related compounds, see: Choudhury *et al.* (2004 ▶); Abid *et al.* (2008 ▶).
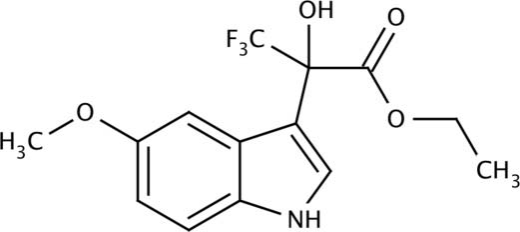


## Experimental

### 

#### Crystal data

C_14_H_14_F_3_NO_4_
*M* *_r_* = 317.26
Monoclinic, 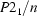

*a* = 9.6277 (4) Å
*b* = 15.9760 (6) Å
*c* = 9.9738 (4) Å
β = 109.314 (5)°
*V* = 1447.75 (10) Å^3^
*Z* = 4
Cu *K*α radiation
μ = 1.15 mm^−1^
*T* = 293 K
0.55 × 0.45 × 0.40 mm

#### Data collection

Oxford Diffraction Xcalibur Ruby CCD diffractometer
Absorption correction: multi-scan (*CrysAlis PRO*; Oxford Diffraction, 2009 ▶) *T*_min_ = 0.782, *T*_max_ = 1.000
5738 measured reflections
2911 independent reflections
2319 reflections with *I* > 2σ(*I*)
*R*_int_ = 0.018

#### Refinement

*R*[*F* ^2^ > 2σ(*F* ^2^)] = 0.040
*wR*(*F* ^2^) = 0.116
*S* = 1.06
2911 reflections
234 parameters
H atoms treated by a mixture of independent and constrained refinement
Δρ_max_ = 0.20 e Å^−3^
Δρ_min_ = −0.14 e Å^−3^

### 

Data collection: *CrysAlis PRO* (Oxford Diffraction, 2009 ▶); cell refinement: *CrysAlis PRO*; data reduction: *CrysAlis PRO*; program(s) used to solve structure: *SHELXS97* (Sheldrick, 2008 ▶); program(s) used to refine structure: *SHELXL97* (Sheldrick, 2008 ▶); molecular graphics: *ORTEP-3 for Windows* (Farrugia, 1997 ▶); software used to prepare material for publication: *publCIF* (Westrip, 2010 ▶).

## Supplementary Material

Crystal structure: contains datablock(s) I, global. DOI: 10.1107/S1600536811021489/pk2322sup1.cif

Structure factors: contains datablock(s) I. DOI: 10.1107/S1600536811021489/pk2322Isup2.hkl

Supplementary material file. DOI: 10.1107/S1600536811021489/pk2322Isup3.cml

Additional supplementary materials:  crystallographic information; 3D view; checkCIF report

## Figures and Tables

**Table 1:** Hydrogen-bond geometry (Å, °)

*D*—H⋯*A*	*D*—H	H⋯*A*	*D*⋯*A*	*D*—H⋯*A*
N1—H1*A*⋯O2^i^	0.86 (2)	2.11 (2)	2.9166 (18)	156.8 (18)
O2—H2*B*⋯O3^ii^	0.80 (2)	2.03 (2)	2.7798 (17)	156 (2)

Symmetry codes: (i) 
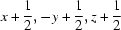
; (ii) 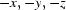.

**Fig. 1.**
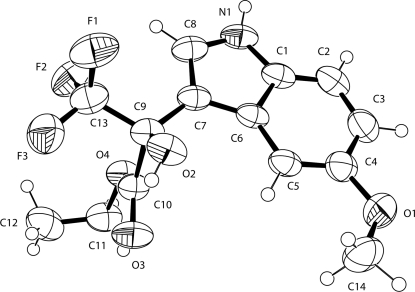
A displacement ellipsoid plot drawn at the 50% probability level with H atoms shown as small spheres of arbitary radius.

**Fig. 2.**
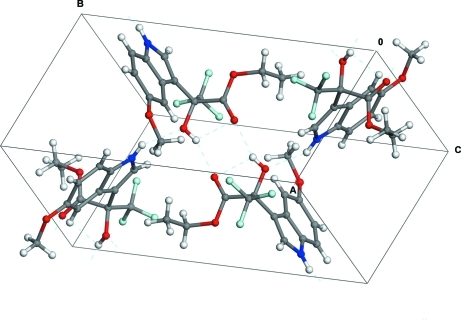
A view of the crystal structure packing, showing part of the hydrogen bonding network.

## supplementary crystallographic information

### Comment

3,3,3-Trifluoropyruvates have been used as efficient fluorinated building blocks in the synthesis of some biologically active trifluoromethylated compounds owing to the unique properties of the trifluoromethyl group, such as high electronegativity, electron density, steric hindrance and its hydrophobic character.  The incorporation of a tertiary α-trifluoromethyl alcohol stereocenter (CF_3_C*(OH)*R*_1_*R*_2_) into heterocycles could provide novel drug candidates with unusual biological activities as a result of the presence of the chiral tertiary α-trifluoromethyl alcohol functionality (Nakamura *et al.* 2008, Abid *et al.*, 2008).

In this study we synthesized the 3,3,3-trifluoropyruvate derivative of indole, which is an analogue of indole alkaloids.  The molecular structure is shown in Fig. 1.

The compound crystallizes as a racemate and the carboxy- and hydroxy- groups are *syn* to each other (torsion angle O2—C9—C10—O3 = 8.1 (2)°. The 3,3,3-trifluoropyruvate fragment is non-coplanar to the plane of the indole [torsion angles C8—C7—C9—C10, C6—C7—C9—C10, C6—C7—C9—O2, C8—C7—C9—O2 are 107.3 (2)°, -69.7 (2)°, 50.0 (2)°, and -133.0 (2)°, respectively)].  The methoxy-, hydroxy- and trifluoromethyl groups deviate from the indole plane by 0.061 (2), 0.854 (1) and 0.165 (2) Å, respectively. The bond distances C—C and C—N in the indole group are in the range 1.369–1.435 Å and 1.358–1.379 Å, respectively.  Due to the concurrent influence of electron-withdrawing groups in the 3,3,3-trifluoropyruvate fragment, the EtO(O)C—C(O)CF_3_ bond is elongated slightly to 1.554 (2) Å.

The fluorine does not readily accept hydrogen bonds and hence behaves differently from chlorine and bromine, but a signifcant number of compounds pack *via* weak interactions involving organic fluorine (Choudhury *et al.*, 2004) and generate different packing motifs *via* F···F, C—H···F and C—F···π interactions. In the presence of a strong acceptor such as C=O, the C—H···O interaction takes priority over C—H···F.   The molecules form inversion-related dimers *via* pairs of intermolecular O—H···O hydrogen bonds.  These dimers are connected by intermolecular N—H···O═C(CF_3_) hydrogen bonds to form a two-dimensional network structure.

### Experimental

A solution of 0.170 g (0.001 mol) of ethyl 3,3,3-trifluoropyruvate in 10 ml of ether was added to a solution of 0.147 g (0.001 mol) of 5-methoxyindole in 10 ml ether, and stirred at room temperature for 24 h.  The reaction was monitored by TLC.  The reaction mixture was then evaporated under reduced pressure and the residue was purified by chromatography on silica  gel (chloroform/ethylacetate=9:1); yield: 0.150 g (47%).  The compound was crystallized from ethanolic solution by slow evaporation, giving colorless prism crystals suitable for X-ray diffraction analysis. ^1^H NMR (300 MHz, dmso-d6): δ 1.11 (3*H*, t, CH_3_); 4.22 (2*H*, q, CH_2_, J_CH3—CH2_ 7,2 Hz); 3.72 (3*H*, s, O—CH_3_), 6.78 (1*H*, d, aromatic, J_6–5_ = 6.9 Hz), 7.17 (1*H*, s, aromatic), 7.32 (1*H*, d, aromatic), 7.37 (2H, s. br., OH, CH), 11.58 (1*H*, s. br., NH); ^19^F NMR (dmso-d6): 3.01 (s, CF_3_); MS (m/*z* 317).

### Refinement

Hydrogen atoms of the NH, OH and hydrogens attached to carbon (except C_Me_) were located in difference Fourier maps and fully refined (including *U*_iso_). Methyl hydrogens were included using a riding model with *U*_iso_(H) values of 1.5 *U*_eq_(C_Me_).

### Crystal data

**Table:**

C_14_H_14_F_3_NO_4_	*F*(000) = 656
*M**_r_* = 317.26	*D*_x_ = 1.456 Mg m^−^^3^
Monoclinic, *P*2_1_/*n*	Cu *K*α radiation, λ = 1.54178 Å
Hall symbol: -P 2yn	Cell parameters from 2319 reflections
*a* = 9.6277 (4) Å	θ = 5.5–75.5°
*b* = 15.9760 (6) Å	µ = 1.15 mm^−^^1^
*c* = 9.9738 (4) Å	*T* = 293 K
β = 109.314 (5)°	Block, colourless
*V* = 1447.75 (10) Å^3^	0.55 × 0.45 × 0.40 mm
*Z* = 4	

### Data collection

**Table:**

Oxford Diffraction Xcalibur Ruby CCD diffractometer	2911 independent reflections
Radiation source: fine-focus sealed tube	2319 reflections with *I* > 2σ(*I*)
graphite	*R*_int_ = 0.018
Detector resolution: 10.2576 pixels mm^-1^	θ_max_ = 75.9°, θ_min_ = 5.5°
ω scans	*h* = −11→12
Absorption correction: multi-scan (*CrysAlis PRO*; Oxford Diffraction, 2009)	*k* = −19→13
*T*_min_ = 0.782, *T*_max_ = 1.000	*l* = −12→12
5738 measured reflections	

### Refinement

**Table:**

Refinement on *F*^2^	Secondary atom site location: difference Fourier map
Least-squares matrix: full	Hydrogen site location: inferred from neighbouring sites
*R*[*F*^2^ > 2σ(*F*^2^)] = 0.040	H atoms treated by a mixture of independent and constrained refinement
*wR*(*F*^2^) = 0.116	*w* = 1/[σ^2^(*F*_o_^2^) + (0.0607*P*)^2^ + 0.1918*P*] where *P* = (*F*_o_^2^ + 2*F*_c_^2^)/3
*S* = 1.06	(Δ/σ)_max_ < 0.001
2911 reflections	Δρ_max_ = 0.20 e Å^−^^3^
234 parameters	Δρ_min_ = −0.14 e Å^−^^3^
0 restraints	Extinction correction: *SHELXL97* (Sheldrick, 2008), Fc^*^=kFc[1+0.001xFc^2^λ^3^/sin(2θ)]^-1/4^
Primary atom site location: structure-invariant direct methods	Extinction coefficient: 0.0092 (10)

### Special details

**Table:**

Geometry. All e.s.d.'s (except the e.s.d. in the dihedral angle between two l.s. planes) are estimated using the full covariance matrix. The cell e.s.d.'s are taken into account individually in the estimation of e.s.d.'s in distances, angles and torsion angles; correlations between e.s.d.'s in cell parameters are only used when they are defined by crystal symmetry. An approximate (isotropic) treatment of cell e.s.d.'s is used for estimating e.s.d.'s involving l.s. planes.Bond distances, angles *etc*. have been calculated using the rounded fractional coordinates. All su's are estimated from the variances of the (full) variance-covariance matrix. The cell e.s.d.'s are taken into account in the estimation of distances, angles and torsion angles
Refinement. Refinement of *F*^2^ against ALL reflections. The weighted *R*-factor *wR* and goodness of fit *S* are based on *F*^2^, conventional *R*-factors *R* are based on *F*, with *F* set to zero for negative *F*^2^. The threshold expression of *F*^2^ > σ(*F*^2^) is used only for calculating *R*-factors(gt) *etc*. and is not relevant to the choice of reflections for refinement. *R*-factors based on *F*^2^ are statistically about twice as large as those based on *F*, and *R*- factors based on ALL data will be even larger.

### Fractional atomic coordinates and isotropic or equivalent isotropic displacement parameters (Å^2^)

**Table:**

	*x*	*y*	*z*	*U*_iso_*/*U*_eq_	
F1	0.24919 (15)	0.21987 (8)	0.04081 (14)	0.0900 (4)	
F2	0.24512 (15)	0.09413 (9)	−0.03770 (13)	0.0863 (4)	
F3	0.42514 (12)	0.13538 (8)	0.14198 (14)	0.0805 (4)	
O1	−0.16538 (14)	0.08409 (10)	0.51949 (16)	0.0773 (4)	
O2	0.04297 (12)	0.12631 (7)	0.09810 (13)	0.0566 (3)	
O3	0.12118 (13)	−0.03385 (7)	0.13702 (13)	0.0622 (3)	
O4	0.34902 (11)	−0.00327 (7)	0.27903 (12)	0.0535 (3)	
N1	0.34543 (16)	0.23072 (9)	0.50886 (17)	0.0590 (4)	
C1	0.22216 (17)	0.19773 (10)	0.53026 (18)	0.0524 (4)	
C2	0.1720 (2)	0.20300 (11)	0.6458 (2)	0.0627 (5)	
C3	0.0439 (2)	0.16245 (13)	0.6367 (2)	0.0658 (5)	
C4	−0.03659 (18)	0.11812 (11)	0.5139 (2)	0.0596 (4)	
C5	0.01333 (17)	0.11079 (10)	0.40028 (19)	0.0528 (4)	
C6	0.14673 (16)	0.15055 (9)	0.40867 (17)	0.0476 (4)	
C7	0.23112 (16)	0.15638 (9)	0.31430 (17)	0.0479 (4)	
C8	0.34965 (18)	0.20619 (10)	0.3800 (2)	0.0556 (4)	
C9	0.19239 (16)	0.11127 (9)	0.17494 (17)	0.0475 (4)	
C10	0.21565 (15)	0.01523 (9)	0.19510 (16)	0.0461 (4)	
C11	0.3900 (2)	−0.09195 (11)	0.2897 (2)	0.0639 (5)	
C12	0.4403 (3)	−0.11618 (14)	0.1696 (3)	0.0826 (6)	
H12A	0.4697	−0.1739	0.1794	0.124*	
H12B	0.5223	−0.0818	0.1702	0.124*	
H12C	0.3613	−0.1085	0.0817	0.124*	
C13	0.2798 (2)	0.14066 (12)	0.0805 (2)	0.0645 (5)	
C14	−0.2605 (2)	0.04673 (16)	0.3932 (2)	0.0810 (6)	
H14A	−0.3506	0.0303	0.4073	0.122*	
H14B	−0.2137	−0.0017	0.3703	0.122*	
H14C	−0.2815	0.0863	0.3166	0.122*	
H1A	0.403 (2)	0.2684 (13)	0.559 (2)	0.066 (6)*	
H2A	0.222 (2)	0.2317 (15)	0.724 (2)	0.079 (6)*	
H2B	0.009 (2)	0.0882 (16)	0.045 (2)	0.079 (7)*	
H3A	0.007 (2)	0.1646 (14)	0.715 (2)	0.075 (6)*	
H5A	−0.0408 (19)	0.0778 (12)	0.3176 (19)	0.056 (5)*	
H8A	0.430 (2)	0.2217 (12)	0.3467 (19)	0.059 (5)*	
H11A	0.304 (2)	−0.1242 (13)	0.292 (2)	0.068 (6)*	
H11B	0.473 (3)	−0.0959 (14)	0.378 (2)	0.079 (6)*	

### Atomic displacement parameters (Å^2^)

**Table:**

	*U*^11^	*U*^22^	*U*^33^	*U*^12^	*U*^13^	*U*^23^
F1	0.1020 (9)	0.0653 (7)	0.0962 (9)	−0.0066 (6)	0.0240 (7)	0.0318 (6)
F2	0.0997 (9)	0.0956 (10)	0.0691 (7)	−0.0147 (7)	0.0352 (6)	−0.0033 (6)
F3	0.0560 (6)	0.0923 (9)	0.0963 (8)	−0.0088 (6)	0.0292 (6)	0.0143 (7)
O1	0.0549 (7)	0.0862 (10)	0.0964 (10)	−0.0112 (7)	0.0323 (7)	−0.0175 (8)
O2	0.0429 (6)	0.0470 (6)	0.0639 (7)	0.0081 (5)	−0.0037 (5)	−0.0038 (6)
O3	0.0518 (6)	0.0471 (6)	0.0710 (7)	−0.0071 (5)	−0.0022 (5)	0.0011 (5)
O4	0.0387 (5)	0.0429 (6)	0.0696 (7)	0.0059 (4)	0.0053 (5)	0.0037 (5)
N1	0.0492 (7)	0.0435 (7)	0.0687 (9)	−0.0106 (6)	−0.0014 (7)	−0.0051 (6)
C1	0.0448 (8)	0.0369 (7)	0.0645 (10)	0.0019 (6)	0.0032 (7)	−0.0040 (7)
C2	0.0611 (10)	0.0519 (10)	0.0645 (11)	0.0052 (8)	0.0066 (9)	−0.0157 (8)
C3	0.0611 (10)	0.0628 (11)	0.0724 (12)	0.0074 (9)	0.0204 (9)	−0.0139 (9)
C4	0.0469 (8)	0.0525 (9)	0.0774 (11)	0.0031 (7)	0.0179 (8)	−0.0085 (8)
C5	0.0420 (8)	0.0442 (8)	0.0648 (10)	−0.0021 (6)	0.0076 (7)	−0.0100 (7)
C6	0.0408 (7)	0.0331 (7)	0.0600 (9)	0.0026 (6)	0.0049 (6)	−0.0033 (6)
C7	0.0404 (7)	0.0358 (7)	0.0594 (9)	0.0005 (6)	0.0055 (6)	0.0032 (6)
C8	0.0437 (8)	0.0459 (9)	0.0673 (10)	−0.0053 (7)	0.0050 (7)	0.0068 (7)
C9	0.0365 (7)	0.0415 (8)	0.0563 (9)	0.0015 (6)	0.0042 (6)	0.0038 (6)
C10	0.0390 (7)	0.0426 (8)	0.0515 (8)	0.0001 (6)	0.0079 (6)	0.0010 (6)
C11	0.0521 (9)	0.0459 (9)	0.0860 (13)	0.0096 (8)	0.0125 (9)	0.0074 (9)
C12	0.0726 (13)	0.0660 (12)	0.1059 (17)	0.0106 (10)	0.0249 (12)	−0.0157 (12)
C13	0.0636 (10)	0.0580 (10)	0.0664 (11)	−0.0046 (8)	0.0139 (8)	0.0111 (9)
C14	0.0487 (10)	0.0883 (15)	0.1008 (16)	−0.0144 (10)	0.0176 (10)	−0.0043 (12)

### Geometric parameters (Å, °)

**Table:**

F1—C13	1.330 (2)	C4—C5	1.375 (3)
F2—C13	1.339 (2)	C5—C6	1.410 (2)
F3—C13	1.332 (2)	C5—H5A	0.972 (18)
O1—C4	1.372 (2)	C6—C7	1.436 (2)
O1—C14	1.421 (3)	C7—C8	1.367 (2)
O2—C9	1.4090 (17)	C7—C9	1.499 (2)
O2—H2B	0.80 (2)	C8—H8A	0.974 (18)
O3—C10	1.1954 (18)	C9—C13	1.530 (3)
O4—C10	1.3137 (17)	C9—C10	1.554 (2)
O4—C11	1.465 (2)	C11—C12	1.484 (3)
N1—C8	1.357 (2)	C11—H11A	0.98 (2)
N1—C1	1.378 (2)	C11—H11B	0.98 (2)
N1—H1A	0.86 (2)	C12—H12A	0.9600
C1—C2	1.391 (3)	C12—H12B	0.9600
C1—C6	1.408 (2)	C12—H12C	0.9600
C2—C3	1.369 (3)	C14—H14A	0.9600
C2—H2A	0.90 (2)	C14—H14B	0.9600
C3—C4	1.405 (3)	C14—H14C	0.9600
C3—H3A	0.97 (2)		
			
C4—O1—C14	117.18 (16)	C7—C9—C13	113.86 (13)
C9—O2—H2B	110.4 (17)	O2—C9—C10	108.44 (12)
C10—O4—C11	116.54 (13)	C7—C9—C10	111.95 (13)
C8—N1—C1	109.48 (14)	C13—C9—C10	107.31 (14)
C8—N1—H1A	122.4 (13)	O3—C10—O4	126.01 (14)
C1—N1—H1A	127.1 (13)	O3—C10—C9	122.07 (13)
N1—C1—C2	131.13 (16)	O4—C10—C9	111.91 (12)
N1—C1—C6	107.25 (16)	O4—C11—C12	110.23 (17)
C2—C1—C6	121.60 (16)	O4—C11—H11A	107.4 (12)
C3—C2—C1	117.93 (17)	C12—C11—H11A	112.8 (12)
C3—C2—H2A	120.9 (14)	O4—C11—H11B	104.3 (13)
C1—C2—H2A	121.2 (14)	C12—C11—H11B	109.3 (13)
C2—C3—C4	121.43 (19)	H11A—C11—H11B	112.5 (17)
C2—C3—H3A	119.9 (13)	C11—C12—H12A	109.5
C4—C3—H3A	118.6 (13)	C11—C12—H12B	109.5
O1—C4—C5	124.53 (16)	H12A—C12—H12B	109.5
O1—C4—C3	114.21 (18)	C11—C12—H12C	109.5
C5—C4—C3	121.26 (17)	H12A—C12—H12C	109.5
C4—C5—C6	118.20 (15)	H12B—C12—H12C	109.5
C4—C5—H5A	120.7 (11)	F1—C13—F3	107.05 (16)
C6—C5—H5A	121.1 (11)	F1—C13—F2	107.46 (16)
C1—C6—C5	119.49 (16)	F3—C13—F2	106.77 (17)
C1—C6—C7	106.70 (14)	F1—C13—C9	111.24 (17)
C5—C6—C7	133.81 (15)	F3—C13—C9	113.91 (15)
C8—C7—C6	106.71 (15)	F2—C13—C9	110.11 (15)
C8—C7—C9	129.49 (16)	O1—C14—H14A	109.5
C6—C7—C9	123.74 (13)	O1—C14—H14B	109.5
N1—C8—C7	109.86 (16)	H14A—C14—H14B	109.5
N1—C8—H8A	121.8 (11)	O1—C14—H14C	109.5
C7—C8—H8A	128.3 (11)	H14A—C14—H14C	109.5
O2—C9—C7	108.57 (13)	H14B—C14—H14C	109.5
O2—C9—C13	106.46 (13)		
			
C8—N1—C1—C2	178.10 (17)	C8—C7—C9—O2	−133.00 (17)
C8—N1—C1—C6	0.03 (18)	C6—C7—C9—O2	50.01 (18)
N1—C1—C2—C3	−179.49 (18)	C8—C7—C9—C13	−14.6 (2)
C6—C1—C2—C3	−1.7 (3)	C6—C7—C9—C13	168.40 (14)
C1—C2—C3—C4	−1.2 (3)	C8—C7—C9—C10	107.32 (18)
C14—O1—C4—C5	−7.2 (3)	C6—C7—C9—C10	−69.67 (18)
C14—O1—C4—C3	172.94 (18)	C11—O4—C10—O3	7.0 (3)
C2—C3—C4—O1	−177.34 (18)	C11—O4—C10—C9	−171.67 (15)
C2—C3—C4—C5	2.8 (3)	O2—C9—C10—O3	8.1 (2)
O1—C4—C5—C6	178.83 (16)	C7—C9—C10—O3	127.84 (17)
C3—C4—C5—C6	−1.3 (3)	C13—C9—C10—O3	−106.54 (18)
N1—C1—C6—C5	−178.63 (14)	O2—C9—C10—O4	−173.15 (13)
C2—C1—C6—C5	3.1 (2)	C7—C9—C10—O4	−53.40 (18)
N1—C1—C6—C7	0.41 (17)	C13—C9—C10—O4	72.22 (17)
C2—C1—C6—C7	−177.88 (14)	C10—O4—C11—C12	83.2 (2)
C4—C5—C6—C1	−1.5 (2)	O2—C9—C13—F1	54.98 (18)
C4—C5—C6—C7	179.74 (16)	C7—C9—C13—F1	−64.61 (19)
C1—C6—C7—C8	−0.69 (17)	C10—C9—C13—F1	170.92 (14)
C5—C6—C7—C8	178.15 (17)	O2—C9—C13—F3	176.06 (15)
C1—C6—C7—C9	176.89 (13)	C7—C9—C13—F3	56.5 (2)
C5—C6—C7—C9	−4.3 (3)	C10—C9—C13—F3	−67.99 (19)
C1—N1—C8—C7	−0.48 (19)	O2—C9—C13—F2	−64.04 (18)
C6—C7—C8—N1	0.73 (18)	C7—C9—C13—F2	176.37 (13)
C9—C7—C8—N1	−176.66 (14)	C10—C9—C13—F2	51.91 (18)

### Hydrogen-bond geometry (Å, °)

**Table:**

*D*—H···*A*	*D*—H	H···*A*	*D*···*A*	*D*—H···*A*
N1—H1A···O2^i^	0.86 (2)	2.11 (2)	2.9166 (18)	156.8 (18)
O2—H2B···O3^ii^	0.80 (2)	2.03 (2)	2.7798 (17)	156 (2)

Symmetry codes: (i) *x*+1/2, −*y*+1/2, *z*+1/2; (ii) −*x*, −*y*, −*z*.
